# Regional brain volume changes in Hakim’s disease versus Alzheimer’s and mild cognitive impairment

**DOI:** 10.1093/braincomms/fcaf122

**Published:** 2025-03-26

**Authors:** Shigeki Yamada, Takuya Yuzawa, Hirotaka Ito, Chifumi Iseki, Toshiyuki Kondo, Tomoyasu Yamanaka, Motoki Tanikawa, Tomohiro Otani, Satoshi Ii, Yasuyuki Ohta, Yoshiyuki Watanabe, Shigeo Wada, Marie Oshima, Mitsuhito Mase

**Affiliations:** Department of Neurosurgery, Nagoya City University Graduate School of Medical Science, Aichi, 467-8601, Japan; Interfaculty Initiative in Information Studies/Institute of Industrial Science, The University of Tokyo, Tokyo, 153-8505, Japan; Medical System Research & Development Center, FUJIFILM Corporation, Tokyo, 107-0052, Japan; Medical System Research & Development Center, FUJIFILM Corporation, Tokyo, 107-0052, Japan; Department of Behavioral Neurology and Cognitive Neuroscience, Tohoku University Graduate School of Medicine, Sendai, Miyagi, 980-8574, Japan; Division of Neurology and Clinical Neuroscience, Department of Internal Medicine III, Yamagata University School of Medicine, Yamagata, 990-9585, Japan; Division of Neurology and Clinical Neuroscience, Department of Internal Medicine III, Yamagata University School of Medicine, Yamagata, 990-9585, Japan; Department of Neurosurgery, Nagoya City University Graduate School of Medical Science, Aichi, 467-8601, Japan; Department of Neurosurgery, Nagoya City University Graduate School of Medical Science, Aichi, 467-8601, Japan; Department of Mechanical Science and Bioengineering, Graduate School of Engineering Science, Osaka University, Osaka, 560-8531, Japan; Department of Mechanical Engineering, School of Engineering, Institute of Science Tokyo, Tokyo, 145-0061, Japan; Division of Neurology and Clinical Neuroscience, Department of Internal Medicine III, Yamagata University School of Medicine, Yamagata, 990-9585, Japan; Department of Radiology, Shiga University of Medical Science, Shiga, 520-2192, Japan; Department of Mechanical Science and Bioengineering, Graduate School of Engineering Science, Osaka University, Osaka, 560-8531, Japan; Interfaculty Initiative in Information Studies/Institute of Industrial Science, The University of Tokyo, Tokyo, 153-8505, Japan; Department of Neurosurgery, Nagoya City University Graduate School of Medical Science, Aichi, 467-8601, Japan

**Keywords:** idiopathic normal-pressure hydrocephalus, Hakim’s disease, Alzheimer’s disease, mild cognitive impairment, artificial intelligence

## Abstract

Idiopathic normal-pressure hydrocephalus (Hakim’s disease) is characterized by ventricular enlargement and disproportionately enlarged subarachnoid space hydrocephalus, leading to localized brain deformation. Differentiating regional brain volume changes in Hakim’s disease from those in Alzheimer’s disease, Hakim’s disease with Alzheimer’s disease, and mild cognitive impairment provides insights into disease-specific mechanisms. This study aimed to identify disease-specific patterns of brain volume changes in Hakim’s disease, Alzheimer’s disease, Hakim’s disease with Alzheimer’s disease, and mild cognitive impairment and compare them with those in cognitively healthy individuals using an advanced artificial intelligence-based brain segmentation tool.

The study included 970 participants, comprising 52 patients with Hakim’s disease, 256 with Alzheimer’s disease, 25 with Hakim’s disease with Alzheimer’s disease, 163 with mild cognitive impairment, and 474 healthy controls. The intracranial spaces were segmented into 100 brain and 7 CSF subregions from 3D T1-weighted MRIs using brain subregion analysis. The volume ratios of these regions were compared among the groups using Glass’s Δ, referencing 400 healthy controls aged ≥50 years. Hakim’s disease exhibited significant volume reduction in the supramarginal gyrus of the parietal lobe and the paracentral gyrus of the frontal lobe. Alzheimer’s disease exhibited prominent volume loss in the hippocampus and temporal lobe, particularly in the entorhinal cortex, fusiform gyrus, and inferior temporal gyrus. Hakim’s disease with Alzheimer’s disease showed significant volume reductions in the supramarginal gyrus of the parietal lobe, similar to Hakim’s disease, whereas temporal lobe volumes were relatively preserved compared with those in Alzheimer’s disease. Patients with mild cognitive impairment aged ≥70 years had comparable regional brain volume ratios with healthy controls in the same age group. The Hakim’s disease and Hakim’s disease with Alzheimer’s disease groups were characterized by volume reductions in the frontal and parietal lobes caused by disproportionately enlarged subarachnoid space hydrocephalus-related compression compared with temporal lobe atrophy observed in the Alzheimer’s disease group. These disease-specific morphological changes highlight the need for longitudinal studies to clarify the causes of compression and atrophy.

## Teaser

Yamada *et al*. used a deep learning model to automatically segment 3D T1-weighted MRI images into 100 detailed brain subregions and 7 CSF subregions, discovering that the volume ratios of the supramarginal gyrus and paracentral gyrus were smaller in patients with Hakim's disease (idiopathic normal-pressure hydrocephalus) compared to healthy controls.

## Graphical Abstract

**Graphical Abstract:**
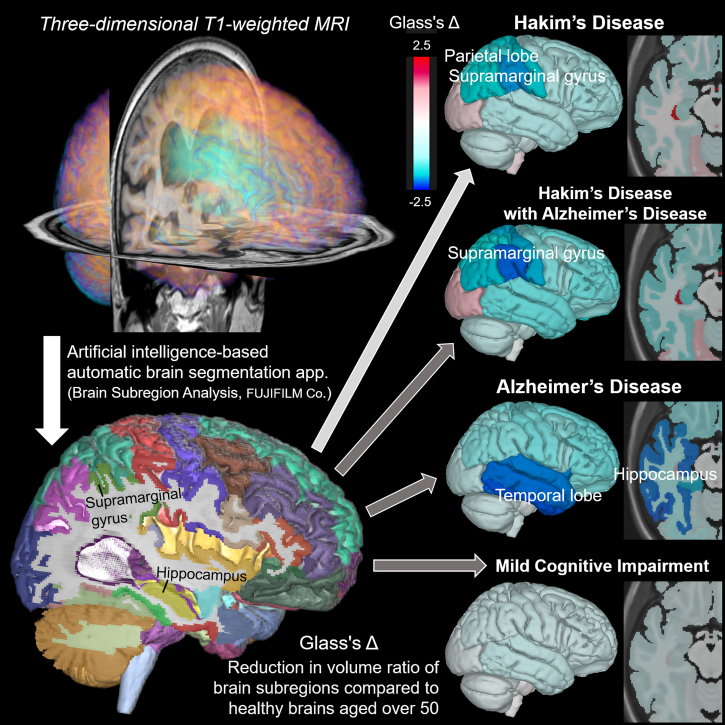

## Introduction

Chronic hydrocephalus in adults, commonly referred to as ‘normal-pressure hydrocephalus’ due to the absence of symptoms associated with intracranial hypertension,^1^ has been classified into idiopathic NPH (iNPH) and secondary NPH in the international (2005)^2,3^ and Japanese (2004)^4^ guidelines for the management of iNPH. Since the publication and subsequent revisions of these guidelines,^3-7^ there has been increasing attention on iNPH, which is characterized by a triad of symptoms: gait disturbance, cognitive dysfunction, and urinary incontinence. Recently, the International Society for Hydrocephalus and Cerebrospinal Fluid Disorders task force team proposed a new classification of seven categories of chronic hydrocephalus in adults and renaming iNPH to Hakim’s disease (HD),^8^ because many experts questioned the term iNPH, i.e. ‘normal pressure’ indicates normal intracranial pressure and ‘idiopathic’ implies unknown causes. HD (or iNPH) is characterized by the chronic accumulation of CSF, and the ventricles are gradually increased and expanded in an upward (cranial) direction.^9^ Furthermore, the Sylvian fissures and basal cisterns are enlarged, causing the brain and subarachnoid spaces to be compressed and displaced towards the cranial midline.^10-12^ There is a shift and narrowing of the higher convexity region, particularly the median sulcus and subarachnoid space.^13-15^ This specific CSF distribution is known as disproportionately enlarged subarachnoid space hydrocephalus (DESH).^12,16-19^ In DESH, the brain surrounding the ventricles is compressed and shrunken; however, it remains unclear which specific regions were most affected by this localized compression and deformation. Kang *et al*. reported abnormal cortical thickening in the parietal, frontal, and occipital lobes in patients with iNPH (HD) compared to healthy controls and patients with Alzheimer’s disease (AD), possibly due to incorrect segmentation caused by morphological changes in DESH.^20^ Bianco *et al*. observed cortical thinning and volume reduction in the frontal, temporal, and cingulate gyri, as well as increased cortical thickness in the superior parietal gyrus in patients with iNPH (HD) regions using FreeSurfer version 6.^21^ However, Del Giovane *et al*. found that FreeSurfer version 7.3.2 often failed to automatically segment brains in iNPH (HD) due to DESH-related deformations, requiring extensive manual corrections.^22^ These previous studies on brain volume and cortical thickness in iNPH (HD) suggest that traditional automated brain region analyses have been challenging due to the highly deformed brains in DESH. We have previously reported on an artificial intelligence-based automatic brain segmentation application developed in 2020, named Brain Subregion Analysis, on an independent 3D volume analyzer workstation (SYNAPSE 3D; FUJIFILM Corporation) from 3D T1-weighted magnetic resonance imaging (MRI). Initially, this application could divide the brain into 21 subregions and the CSF into 5 regions.^23^ In 2024, this application was upgraded and could automatically and more accurately segment the brain into 100 subregions and the CSF into seven regions.^24^ We aimed to investigate which part of the brain in patients with HD (iNPH) decreased its volume ratio in reference to the intracranial space using this updated application. However, the observation of longitudinal changes using 3D MRI before any interventions is challenging. Therefore, patients before CSF shunt surgery were compared with age-matched healthy controls. Furthermore, patients with Ad, the co-occurrence of HD and AD (HD + AD), and mild cognitive impairment (MCI) were also compared with cognitively healthy individuals aged ≥50 years.

## Materials and methods

### Ethical approvals

The study design and protocol of this prospective observational study were approved by the Ethics Committees for Human Research of our institutions (IRB Number: 60-22-0083, 2022-73, R2019-227). After explaining the aim of this study and the potential for the detection of diseases in the brain, the healthy volunteers provided written informed consent and underwent MRI examinations. The patients’ MRI data were obtained using an opt-out method after their personal information was anonymized in a linkable manner. The study was conducted according to the approved guidelines of the Declaration of Helsinki.

### Study population

The dataset of 3D T1-weighted MRIs in this study that included participants was previously reported.^19^ In brief, the 3D T1-weighted MRIs were magnetization prepared rapid gradient echo sequences on 1.5- or 3-T MRI machines made by GE Healthcare (USA), Siemens AG (Germany), and Philips (The Netherlands) at the collaborating hospitals; the three collaborating hospitals and volunteers from two cohorts, with data collected retrospectively and continuously. The inclusion criteria were as follows: individuals with suspected brain disorders who had not undergone brain surgery and had undergone a 3D T1-weighted MRI. The exclusion criteria were as follows: chronic hydrocephalus in adults in six categories, except for HD, the presence of space-occupying lesions in the brain, and imaging data that could not be processed using the CSF Space Analysis and Brain Subregion Analysis applications on an independent 3D volume analyzer workstation (SYNAPSE 3D; FUJIFILM Corporation). Nine individuals were excluded from the extraction of all 100 brain subregions on the Brain Subregion Analysis application for the reasons of motion artefact or some imaging issues, two individuals were excluded for incidental chronic subdural haematomas, and one individual was excluded due to finding a brain tumour. Furthermore, 24 patients with Lewy body dementia; seven with frontotemporal dementia; 81 with mixed dementia; 6 with neurodegenerative diseases, including Parkinson’s disease and corticobasal degeneration; 10 with mental disorders; and five with stroke were excluded. In addition, 3 patients with Ad, five with MCI, and 12 healthy individuals who were diagnosed with DESH on MRI, but did not exhibit typical symptoms of gait disturbance, cognitive impairment, or urinary incontinence, were classified as asymptomatic ventriculomegaly with iNPH features on MRI.^25^ As asymptomatic ventriculomegaly with iNPH features on MRI patients were not diagnosed as HD (iNPH), they were excluded from this study. The diagnosis of HD or HD + Ad in this study was based on the latest Japanese guidelines for the management of iNPH.^7^ Finally, this study included 77 patients with HD, 256 with Ad, and 163 with MCI. Of the 77 patients with HD, 25 were concurrently diagnosed with Ad (HD + Ad). Overall, 56 patients with HD (73%) underwent the CSF tap test, whereas 41 (53%) underwent CSF shunt surgery, and their symptoms improved by ≥1 point based on the results of the modified Rankin scale and/or the Japanese iNPH Grading Scale.^7^ Ad and MCI were diagnosed by neurologists according to recent criteria established by the National Institute of Neurological and Communicative Disorders and Stroke and the Alzheimer’s Disease and Related Disorders Association.^26-28^

### Data processing, segmentation, and measurement

The Brain Subregion Analysis application on an independent 3D volume analyzer workstation (SYNAPSE 3D; FUJIFILM Corporation) was approved as a medical device by the Pharmaceuticals and Medical Devices Agency of Japan in 2020. In 2023, this application was then upgraded to enable automatic segmentation of the brain into 107 subregions (3D videos of brain subregion analysis are included in the Supplementary materials). Using this application, the brain on the 3D T1-weighted sequence was automatically segmented, and the segmented volumes were quantified within 1 min into 100 brain subregions and 7 CSF spaces: the bilateral upper parts and inferior horns of the lateral ventricles, third ventricle, fourth ventricle, and subarachnoid spaces. The segmentation accuracy was compared with two major atlas-based volumetry methods—statistical parametric mapping and FreeSurfer—in a previous study.^24^ The results confirmed that the brain subregion analysis had the best performance in terms of both repeatability and reproducibility among the three applications. In this study, the cortical grey matter was defined as the combined region of the frontal, temporal, parietal, occipital, and insular cortex, and the subcortical grey matter was defined as the combined region of the hippocampus, basal ganglia, and brainstem.

### Statistical analysis

The cognitively healthy volunteers were divided into seven subgroups based on their ages at the time of MRI examination: 20, 30, 40, 50, 60, 70 s, and ≥80 years. The volume ratios of the segmented brain and CSF spaces, which were defined as the volume divided by intracranial volume, among the four subgroups—HD, Ad, HD + Ad, and MCI—were compared with those of the healthy controls aged ≥50 years using Glass’s Δ,^29^ which was calculated as the difference between the mean volume ratio of the patient group and that of the controls, divided by the standard deviation (SD) of the controls. The frontal lobe cortex was subdivided into 11 subregions, the temporal lobe cortex into 9 subregions, the parietal lobe cortex into 5 subregions, and the occipital lobe cortex into 4 subregions. To maximize the visualization of volume ratio differences compared to healthy control, the final Glass’s Δ for these detailed subregions in this study were derived by incorporating the weighted Glass’s Δ from the mid-subregions, such as the frontal, temporal, parietal, and occipital lobe cortex. In particular, Glass’s Δ for each subregion of the frontal lobe cortex was calculated by adding a value obtained by dividing Glass’s Δ of the entire frontal lobe cortex by 11. Similarly, Glass’s Δ for each subregion of the temporal lobe cortex was calculated by adding a value obtained by dividing Glass’s Δ of the entire temporal lobe cortex by nine. These adjustments yielded the final Glass’s Δ for each subregion. The Kruskal–Wallis rank-sum test was used to compare the mean volume ratios of the segmented brain among the seven age subgroups. The *χ*² test was used to compare the proportions of the groups. Statistical significance was assumed at a probability (*P*) value of <0.05. However, for the *t*-tests comparing the mean volume ratios of the 100 detailed brain subregions between the four disease groups and the healthy control group, the significance level was adjusted to *P* < 0.0005 using the Bonferroni correction. Statistical analyses were performed using R (version 4.2.3; The R Foundation for Statistical Computing; http://www.R-project.org).

## Results

### Clinical characteristics and sex differences

This study enrolled 970 participants from three collaborating hospitals and two cohorts in a retrospective, consecutive case manner. The participants comprised 52 patients diagnosed with HD, 256 with Ad, 25 with HD + Ad, 163 with MCI, 400 cognitively healthy controls aged ≥50 years, and 74 cognitively healthy individuals aged <50 years (Table 1). All patients with HD had a DESH feature and any symptoms of gait disturbance, cognitive impairment, and urinary incontinence. In total, 98 participants (10%) were evaluated to have DESH, 164 (17%) with ventriculomegaly, 93 (9.6%) with tightening of the sulci at the high convexity and midline, and 199 (21%) with Sylvian fissure dilatation.

**Table 1:** Clinical characteristics of the study population

	HD	HD + Ad	Ad	MCI	Healthy 50+	Healthy 50-
Total	52	25	256	163	400	74
Male	29 (55.8%)	15 (60%)	87(34.0%)	81 (49.7%)	167 (41.8%)	28 (37.8%)
Female	23 (44.2%)	10 (40%)	169 (66.0%)	82 (50.3%)	233 (58.3%)	46 (62.2%)
DESH	52 (100%)	25 (100%)	3 (1.2%)	5 (3.1%)	13 (3.3%)	0
VD	51 (98.1%)	25 (100%)	45 (17.6%)	17 (10.4%)	26 (6.5%)	0
THC	50 (96.2%)	24 (96.0%)	3 (1.2%)	2 (1.2%)	14 (3.5%)	0
SFD	39 (75.0%)	24 (96.0%)	64 (25.0%)	31 (19.0%)	41 (10.3%)	0
Age, Range	60–92	62–96	61–99	56–92	50–99	21–49
Age, Mean ± SD	77.2 ± 6.9	81.4 ± 7.2	79.4 ± 7	78.9 ± 6.5	74.6 ± 8.4	34.6 ± 8.3
Total CSF (mL)	334.6 ± 45.0	381.9 ± 71.4	409 ± 67.3	332.9 ± 73.2	304.2 ± 61.1	268.8 ± 47.6
Total ventricle (mL)	135.1 ± 33.1	123.5 ± 30.8	71 ± 22.8	59.1 ± 20.5	49.6 ± 20.5	22.6 ± 12.1
HCS	14.5 ± 8.5	16.4 ± 7.0	48.7 ± 13.8	40.0 ± 14.3	38.8 ± 13.6	41.5 ± 8.9
Syl + Bc	68.1 ± 20.1	80.6 ± 23.7	64.8 ± 14.9	54.8 ± 12.5	50.6 ± 11.5	41.7 ± 7.3
DESH index, Mean ± SD	21.1 ± 17.2	17.1 ± 13.9	3.1 ± 1.4	3.3 ± 2.0	3.1 ± 2.6	1.6 ± 0.4
Venthi index, Mean ± SD	14.6 ± 13.3	11.2 ± 11.1	1.6 ± 0.9	1.8 ± 1.3	1.6 ± 1.7	0.6 ± 0.3
Sylhi index, Mean ± SD	6.5 ± 4.2	5.9 ± 3.1	1.4 ± 0.6	1.5 ± 0.8	1.5 ± 1	1 ± 0.2

HD, Hakim’s disease; Ad, Alzheimer’s disease; HD + Ad, Hakim’s disease with Alzheimer’s disease; MCI, mild cognitive impairment; healthy 50+, cognitively healthy individuals aged ≥50 years; healthy 50-, cognitively healthy individuals under 50 years old; DESH, disproportionately enlarged subarachnoid space hydrocephalus; VD, ventricular dilatation; THC, tightened sulci in the high convexities; SFD, Sylvian fissure dilation; CSF, cerebrospinal fluid; HCS, high-convexity part of the subarachnoid space; Syl + Bc, Sylvian fissure and basal cistern; DESH index, total ventricular volume, and the Syl + Bc volume divided by the HCS volume; Venthi index, ventricular volume divided by the HCS volume; Sylhi index, Syl + Bc volume divided by the HCS volume.

### Ageing-related reduction of regional brain volume and cerebrospinal fluid spaces’ enlargement

**Figure 1:**
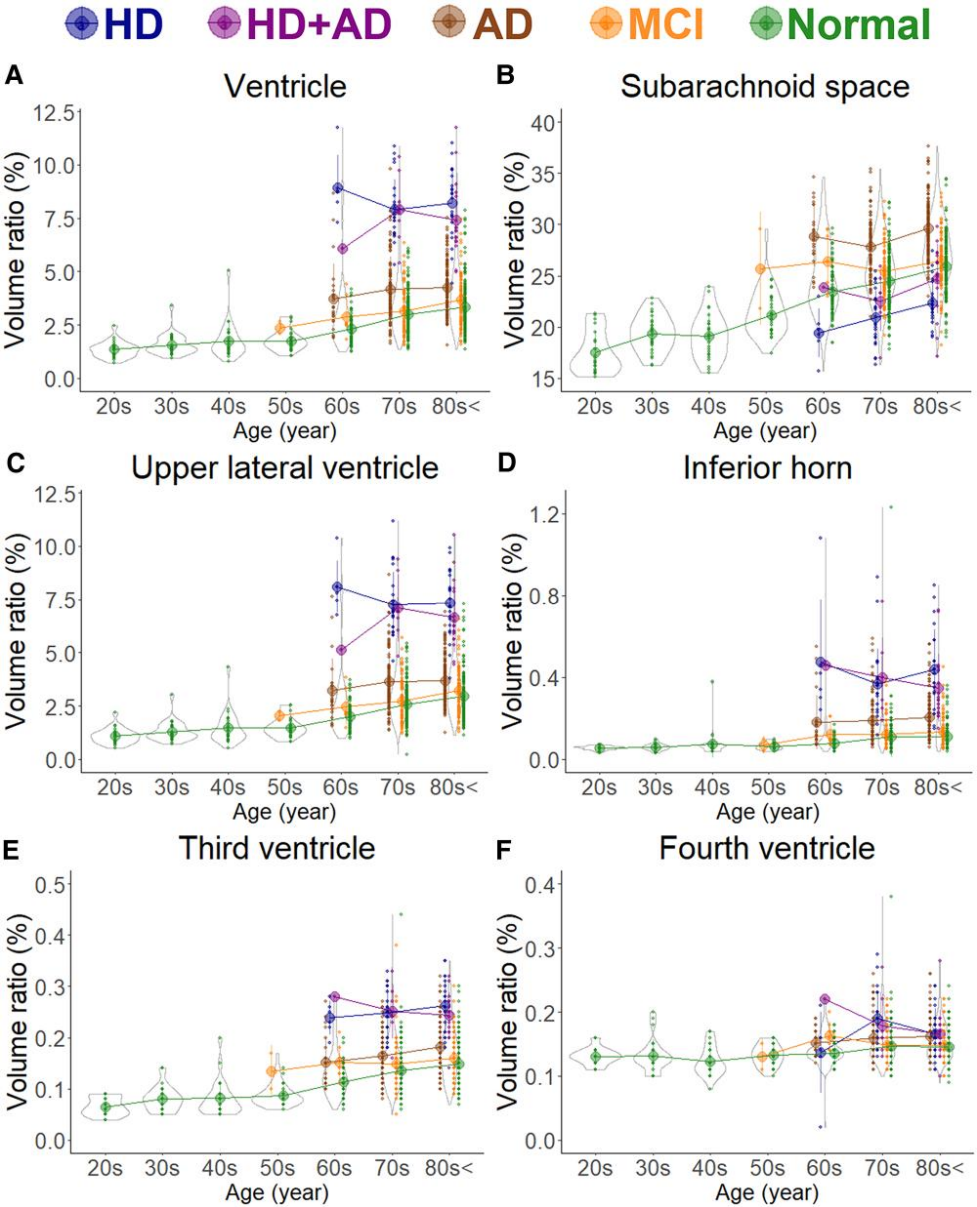
**Segmented region volume ratio of cerebrospinal fluid spaces.** Each graph has violin plots for the distribution of the segmented volume ratio and a circle with line graphs for the mean volume ratio in each decade stratified according to disease. Hakim’s disease (HD, *N* = 52), Alzheimer’s disease (AD, *N* = 256), HD with AD (HD + AD, *N* = 25), mild cognitive impairment (MCI, *N* = 163), and normal volunteers (*N* = 474). (**A**) Ventricle; (**B**) Subarachnoid space; (**C**) Upper part of lateral ventricle; (**D**) Inferior horn of lateral ventricle; (**E**) Third ventricle; and (**F**) Fourth ventricle.

**Figure 2:**
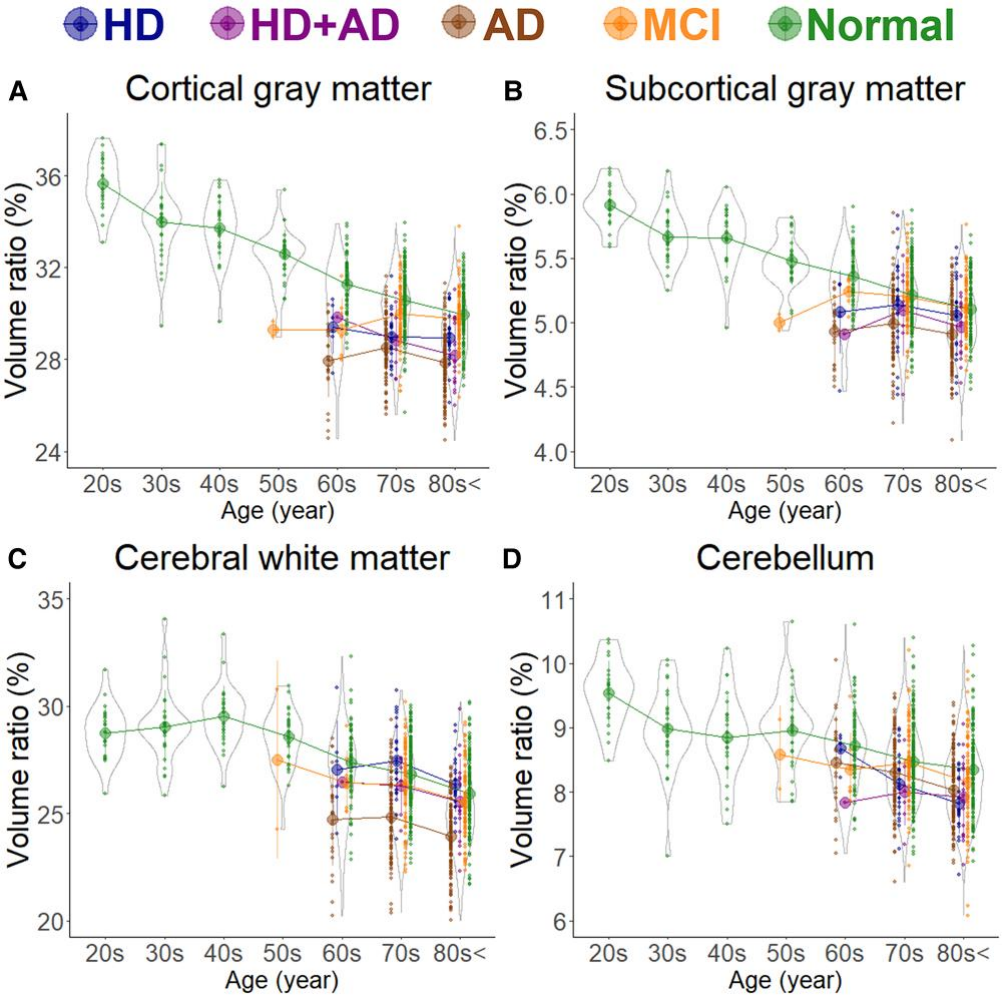
**Segmented region volume ratio of large subregions.** Each graph has violin plots for the distribution of the segmented volume ratio and a circle with line graphs for the mean volume ratio in each decade stratified according to disease. Hakim’s disease (HD, *N* = 52), Alzheimer’s disease (AD, *N* = 256), HD with AD (HD + AD, *N* = 25), mild cognitive impairment (MCI, *N* = 163), and normal volunteers (*N* = 474). (**A**) Cortical grey matter; (**B**) Subcortical grey matter; (**C**) Cerebral white matter; and (**D**) Cerebellum.

**Figure 3:**
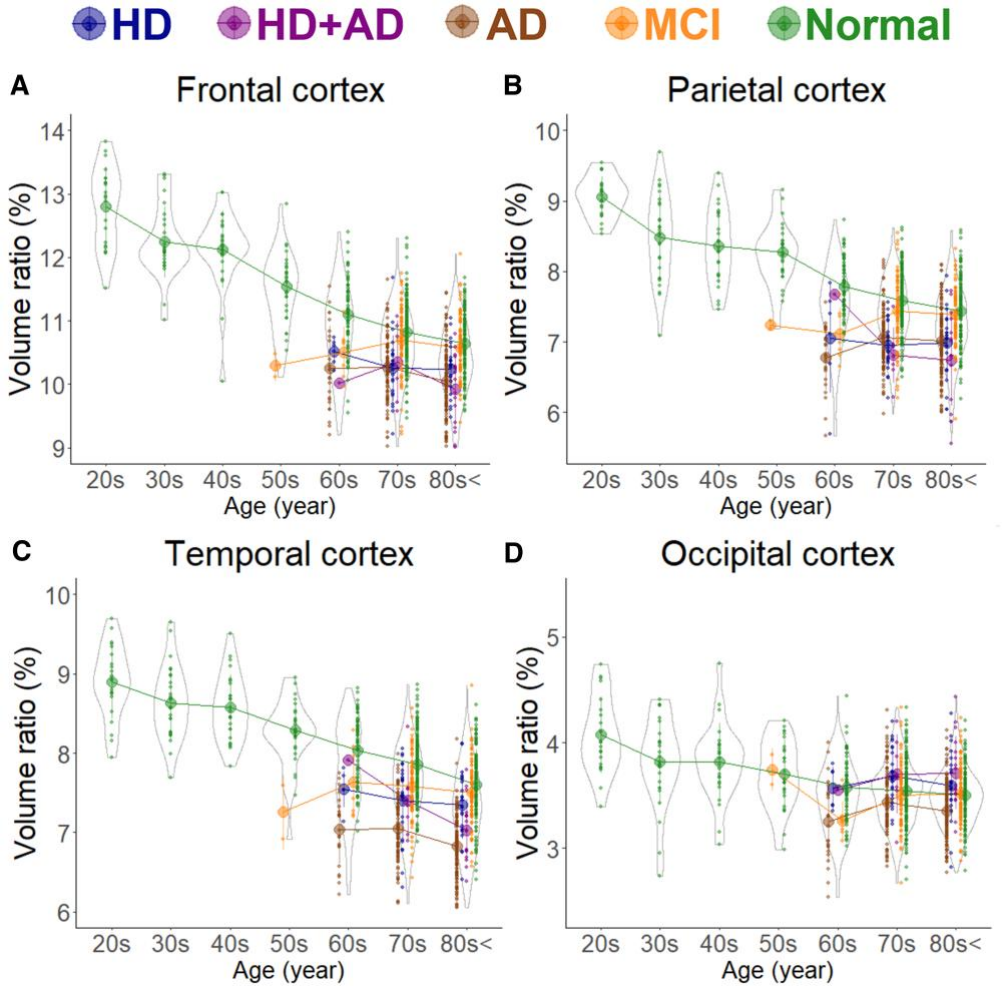
**Segmented region volume ratio of mid-subregions.** Each graph has violin plots for the distribution of the segmented volume ratio and a circle with line graphs for the mean volume ratio in each decade stratified according to disease. Hakim’s disease (HD, *N* = 52), Alzheimer’s disease (AD, *N* = 256), HD with AD (HD + AD, *N* = 25), mild cognitive impairment (MCI, *N* = 163), and normal volunteers (*N* = 474). (**A**) Frontal cortex; (**B**) Parietal cortex; (**C**) Temporal cortex; and (**D**) Occipital cortex.

In healthy individuals, as age increased, the brain volume ratio decreased, accompanied by compensatory increases in the volume ratios of the subarachnoid space and ventricles (Fig. 1). The cortical and subcortical grey matter volume ratio decreased linearly with ageing (Fig. 2A and B), whereas the white matter volume ratio slightly increased until the 40 s and then decreased after the 50 s (Fig. 2C). Among healthy individuals, the frontal lobe, which had the largest mean volume ratio, decreased from 12.8% in the 20 s to 10.6% in the 80 s or older, showing the largest reduction of 2.2% (Fig. 3A). The occipital lobe, which had the smallest volume ratio, decreased from 4.1% in the 20 s to 3.5% in the 80 s or older, with a smaller reduction of 0.6% (Fig. 3D).

### Reduction in regional brain volume in various diseases

**Figure 4:**
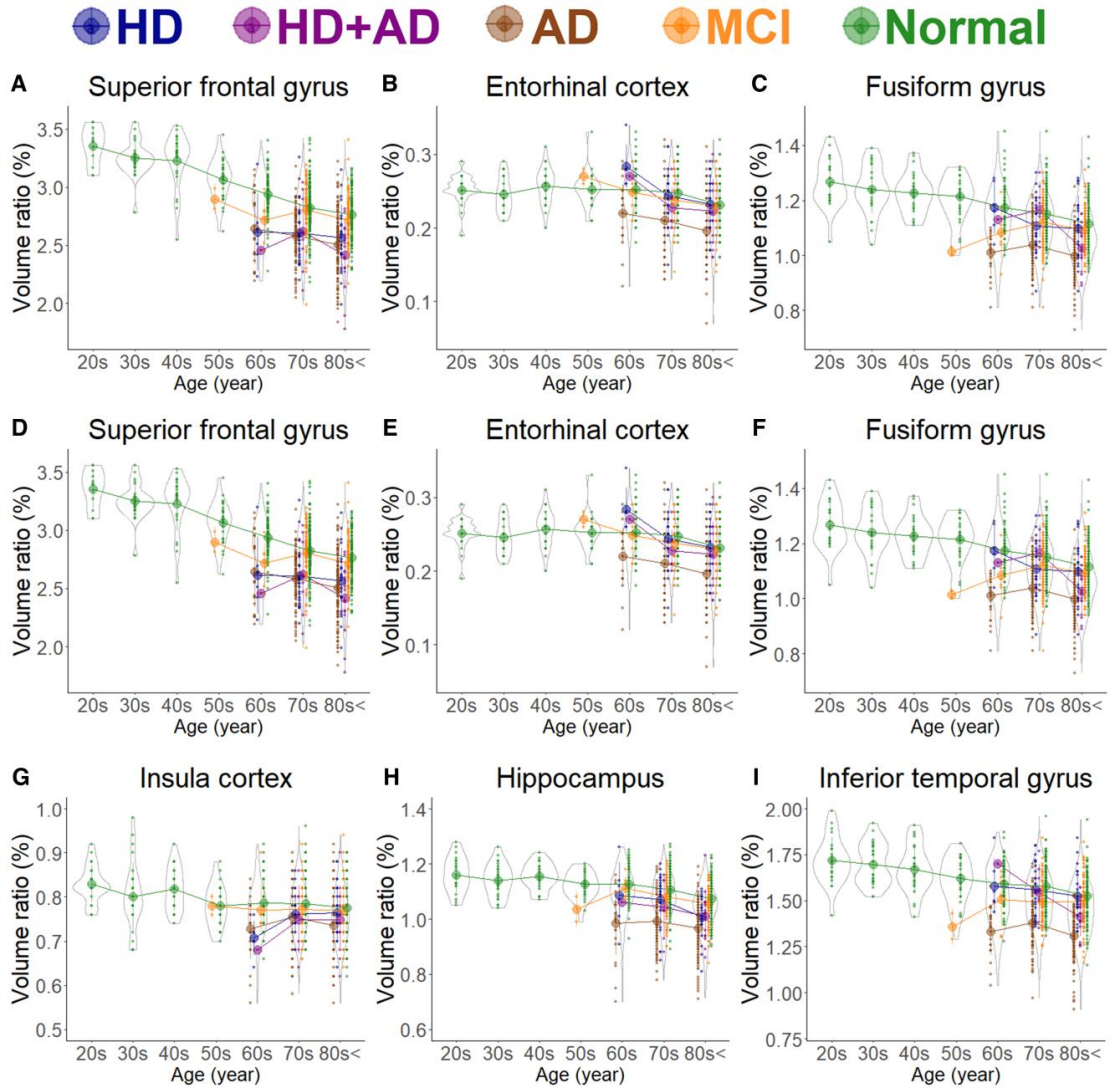
**Segmented region volume ratio of specific brain subregions.** Each graph has violin plots for the distribution of the segmented volume ratio and a circle with line graphs for the mean volume ratio in each decade stratified according to disease. Hakim’s disease (HD, *N* = 52), Alzheimer’s disease (AD, *N* = 256), HD with AD (HD + AD , *N* = 25), mild cognitive impairment (MCI, N = 163), and normal volunteers (*N* = 474). (**A**) Supramarginal gyrus; (**B**) Pars triangularis; (**C**) Paracentral gyrus; (**D**) Superior frontal gyrus; (**E**) Entorhinal cortex; (**F**) Fusiform gyrus; (**G**) Insula cortex; (**H**) Hippocampus; and (**I**) Inferior temporal gyrus.

As shown in Fig. 1B, the subarachnoid space volume ratio was the largest in the Ad group across all ages, although the ventricular volume ratio was slightly larger than that in the MCI group (Fig. 1A). In the HD and HD + Ad groups, the ventricular volume ratio was markedly larger across all ages (Fig. 1A), with enlargement of the lateral and third ventricles (Fig. 1C–E), although the fourth ventricle exhibited no significant differences (Fig. 1F). Among the five groups, the volume ratios of cortical and subcortical grey matter and cerebral white matter were smallest in the Ad group across all ages, largest in healthy controls, and intermediate in the MCI, HD, and HD + Ad groups (Fig. 2A–C). Specifically, the mean volume ratios of cortical grey matter in individuals aged 60 years or younger were significantly smaller in the MCI group than in age-matched healthy controls, whereas in individuals aged 70 years or older, the ratios were similar between the MCI group and healthy controls (Fig. 2A). The mean volume ratio of subcortical grey matter in individuals aged 50 years or younger was significantly smaller in the MCI group than in age-matched healthy controls, whereas in individuals aged 60 years or older, the ratios were similar between the two groups (Fig. 2B). The mean volume ratios of cerebral white matter were the smallest in the Ad group across all age ranges (Fig. 2C). While the volume ratio of the cerebellar cortex exhibited a slight decrease with ageing, no disease-specific reduction was observed (Fig. 2D). The Ad group showed significantly reduced volume ratios in the hippocampus (Fig. 4H) and temporal lobe (Fig. 3C), particularly in the entorhinal cortex (Fig. 4E), fusiform gyrus (Fig. 4F), and inferior temporal gyrus (Fig. 4I), which were markedly smaller than those in the other four groups across all ages. In contrast, the HD and HD + Ad groups exhibited significantly lower volume ratios in the supramarginal gyrus (Fig. 4A) of the parietal lobe, pars triangularis (Fig. 4B) and paracentral gyrus (Fig. 4C) of the frontal lobe, and insula cortex (Fig. 4G) of the temporal lobe than the other three groups. The volume ratio in the superior frontal gyrus was similarly lower in the HD, HD + Ad, and Ad groups compared to that in the healthy control and MCI groups (Fig. 4D). Supplementary Figs 1–3 present the distribution of the other subregions according to disease and age. In Supplementary Table 1, the mean volume ratios of the 100 detailed brain subregions were compared among the four disease groups and the healthy control group.

### Glass’s Δ compared with the mean of healthy brains

**Figure 5:**
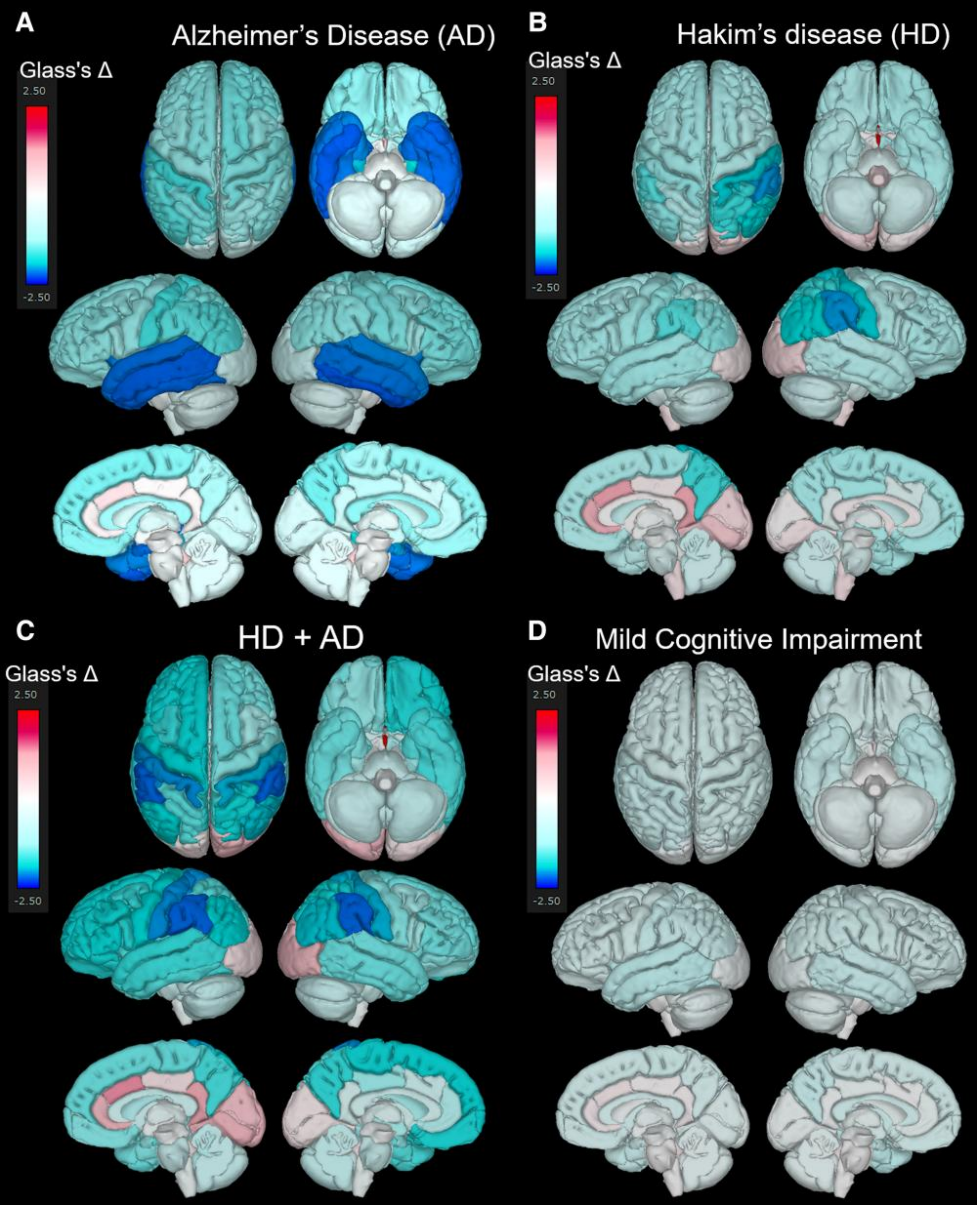
**Superficial distribution of the mean Glass’s Δ.** Superior, inferior, outer lateral, and inner lateral surface views showing the distribution of the mean Glass’s Δ in the Alzheimer’s disease (AD, *N* = 256) (**A**), Hakim’s disease (HD, *N* = 52) (**B**), HD with AD (HD + AD, *N* = 25) (**C**), and mild cognitive impairment groups (*N* = 163) (**D**).

**Figure 6:**
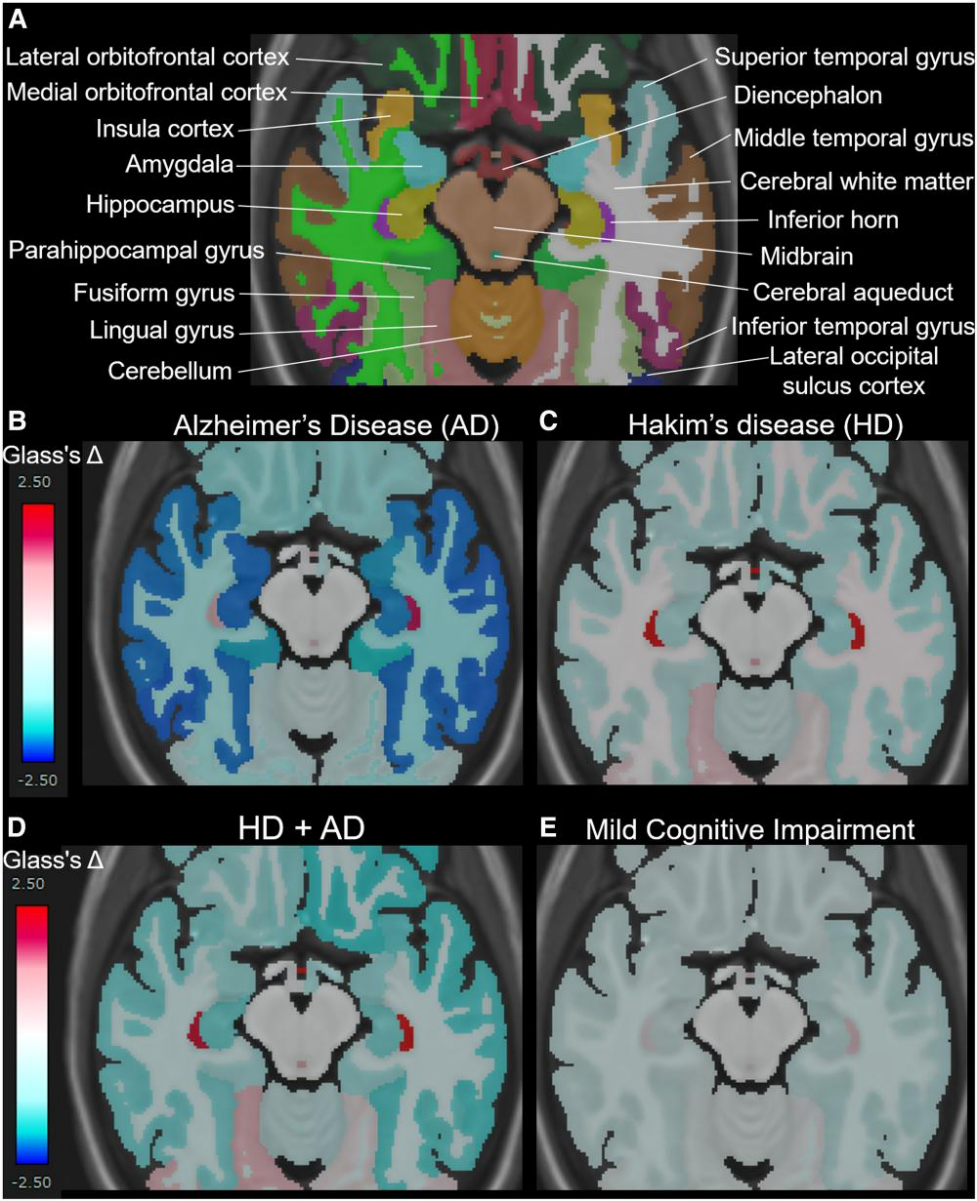
**Mean Glass’s Δ on axial views surrounding the hippocampus and temporal lobe.** A shows the names of each automatically segmented subregion observed within the cross-section. The mean Glass’s Δ in the Alzheimer’s disease (AD, *N* = 256) (**B**), Hakim’s disease (HD, *N* = 52) (**C**), HD with AD (HD + AD, *N* = 25) (**D**), and mild cognitive impairment groups (*N* = 163) (**D**).

Figure 5 presents the mean Glass’s Δ of subregions in the HD, HD + Ad, Ad, and MCI groups, compared with that in the 400 healthy controls aged ≥50 years, which was shown from the superior, inferior, outer lateral, and inner lateral surface views. The Ad group (Fig. 5A) showed pronounced volume loss in the hippocampus and temporal lobe, whereas the HD group (Fig. 5B) exhibited significant volume loss in the parietal lobe, with no obvious volume loss in the temporal lobe. Notably, the HD + Ad group (Fig. 5C) did not show the temporal lobe volume loss observed in the Ad group. Instead, the parietal lobe, particularly the supramarginal gyrus, exhibited a significantly smaller volume ratio than the healthy controls (Fig. 5C). As shown in Fig. 5D, the MCI group also exhibited significantly smaller volume ratios across the whole brain compared to healthy controls; however, no region-specific volume reductions, such as those observed in the temporal lobe of the Ad group, were detected. Axial views focusing on the hippocampus and temporal lobe (Fig. 6) revealed that the volume ratios of the hippocampus and temporal lobe cortex in the HD + Ad group (Fig. 6D) exhibited less pronounced reductions than those in the Ad group (Fig. 6B) but more significant reductions than those in the HD group (Fig. 6C). In contrast, the MCI group (Fig. 6E) exhibited reduced volume ratios across all subregions compared with the healthy controls, without any region displaying a distinct pattern of volume loss similar to that observed in the Ad or HD group.

## Discussion

This study is the first comparative analysis of the regions of local brain compression and deformation caused by enlargement of the ventricles and Sylvian fissure in HD (iNPH), distinguishing it from HD + Ad and comparing it with local brain atrophy in Ad, MCI, and healthy ageing brains. In this study, the most prominent volume reduction in HD and HD + Ad was observed in the supramarginal gyrus of the parietal lobe. No similar findings have been reported, which may have been because HD is characterized by ventriculomegaly and DESH, which cause significant deformation not observed in other diseases.^16^ This often leads to failures when using atlas-based regional segmentation methods. However, our deep learning-based hierarchical brain segmentation method includes training data from cases of HD with DESH, thereby enabling accurate automatic extraction.^24^ In HD, the complexity of symptoms has previously made it challenging to clarify the brain’s functional localization. The volume loss of the supramarginal gyrus in HD and HD + Ad may be influenced by compression due to concurrent enlargement of the lateral ventricles and Sylvian fissure. The supramarginal gyrus is involved in integrating spatial information, allocating attention, and facilitating adaptive behaviours to the surrounding environment,^30,31^ which is consistent with the characteristic cognitive impairments observed in HD.^32,33^ In addition, volume loss in the paracentral gyrus has also been observed in HD, which may be associated with gait disturbance, postural instability, and urinary disturbances, including incontinence.^34^ Freezing of gait, a well-known gait disturbance in Parkinson’s disease,^35^ has been reported to be associated with the paracentral gyrus.^36-39^ However, gait disturbances in patients with HD also exhibit several features similar to those in Parkinson’s disease, such as freezing of the gait and postural instability.^40^ Another important aspect was that the volume loss pattern in HD + Ad differed entirely from that in Ad. HD is characterized by impaired CSF clearance, and it has been suggested that the resulting CSF stagnation facilitates the accumulation of brain waste products, such as amyloid-beta and tau proteins, which may explain the high comorbidity with Ad.^41-44^ In particular, since the discovery of the glymphatic system,^45,46^ HD has gained attention as a disease closely associated with the pathogenesis of neurodegenerative disorders,^47,48^ including Ad. It is believed that improving CSF perfusion has the potential to clear brain waste products, and early therapeutic intervention in HD is expected to reduce the risk of Ad comorbidity.^49^ Notably, the finding that patients with HD + Ad exhibited no significant atrophy in the hippocampus or temporal lobes, but rather prominent atrophy in the posterior lateral part of the parietal lobe, does not contradict this hypothesis. Posterior cortical atrophy is recognized as a variant of Ad, characterized by distinctive patterns of brain atrophy and specific clinical symptoms.^50-52^ Unlike typical Ad, in which atrophy in the temporal lobe is predominant, posterior cortical atrophy involves more pronounced atrophy in the occipital and/or parietal lobes, with relatively minimal involvement of the temporal lobe in the early stages.^53^ This pattern of atrophy leads to a unique presentation of symptoms, such as visual processing, attention deficits, and executive dysfunction, and memory impairment is less prominent. In addition, the symptoms of spatial orientation, visual recognition, and task execution were similar to those in HD and HD + Ad. Based on these findings, we hypothesized that patients diagnosed with HD + Ad may have a pathological condition resembling the posterior cortical atrophy variant of Ad. However, the diagnosis of concurrent Ad in HD was not based on pathology by brain biopsy or amyloid PET or tau PET study but rather on symptoms and findings, such as reduced blood flow patterns in the parietal association cortex, temporal association cortex, posterior cingulate gyrus, and precuneus on single-photon emission computed tomography and elevated phosphorylated tau concentrations in CSF.

This study has some limitations. First, the study design was cross-sectional, involving cognitively healthy individuals with wide-ranging ages and patients diagnosed with HD, HD + Ad, Ad, and MCI at one point. Longitudinal assessments should be ideal to demonstrate brain volume loss with ageing and diseases. Second, whether the observed volume reduction in specific brain subregions in HD, compared with age-matched healthy controls, was primarily attributable to atrophy or compression resulting from morphological changes, such as DESH, remains uncertain. Future investigations, including the evaluation of volume ratio recovery following CSF shunt surgery in patients with HD and HD + Ad, may help distinguish between atrophic and compressive changes, thereby shedding light on the pathological mechanisms underlying these morphological alterations. Finally, all patients in this study were diagnosed with or without Ad or comorbid Ad by neurologists specializing in dementia at each collaborating hospital, based on the diagnostic criteria current at the time of evaluation. While some patients underwent single-photon emission computed tomography or CSF phosphorylated tau measurement, these supplemental tests were not performed on all patients. Furthermore, amyloid or tau PET, as recommended in recent Ad guideline updates,^54,55^ was not conducted.

In conclusion, we found that the patients diagnosed with HD and HD + Ad had significant volume reductions in the supramarginal gyrus of the parietal lobe, pars triangularis and paracentral gyrus of the frontal lobe, and insula cortex of the temporal lobe, which may be caused by compression due to DESH-related morphological changes (e.g. the concurrent enlargement of the lateral ventricles and Sylvian fissure). These regional brain volume alterations specific to HD and HD + Ad differed from the volume reductions in the hippocampus and temporal lobe in Ad. These results highlight the need for longitudinal research to better understand the underlying mechanisms and implications of these morphological alterations.

## Supplementary Material

fcaf122_Supplementary_Data

## Data Availability

The data and results in this study were analyzed using The Brain Subregion Analysis application (FUJIFILM Corporation), which utilized the Alzheimer’s disease Neuroimaging Initiative database (adni.loni.usc.edu) as training data for deep learning. No other publicly available repositories were used. In this study, we did not generate any new code; therefore, sharing data or code is not applicable to this article.
