# Supplementary material for: Regional brain volume changes in Hakim’s disease versus Alzheimer’s and mild cognitive impairment
Source: Brain Commun. 2025 Mar 26;7(2):fcaf122. doi: 10.1093/braincomms/fcaf122 (PMC11997787; doi:10.1093/braincomms/fcaf122)
Supplement: fcaf122_Supplementary_Data [file fcaf122_Supplementary_Data.zip › Supplementary Figures.pdf]

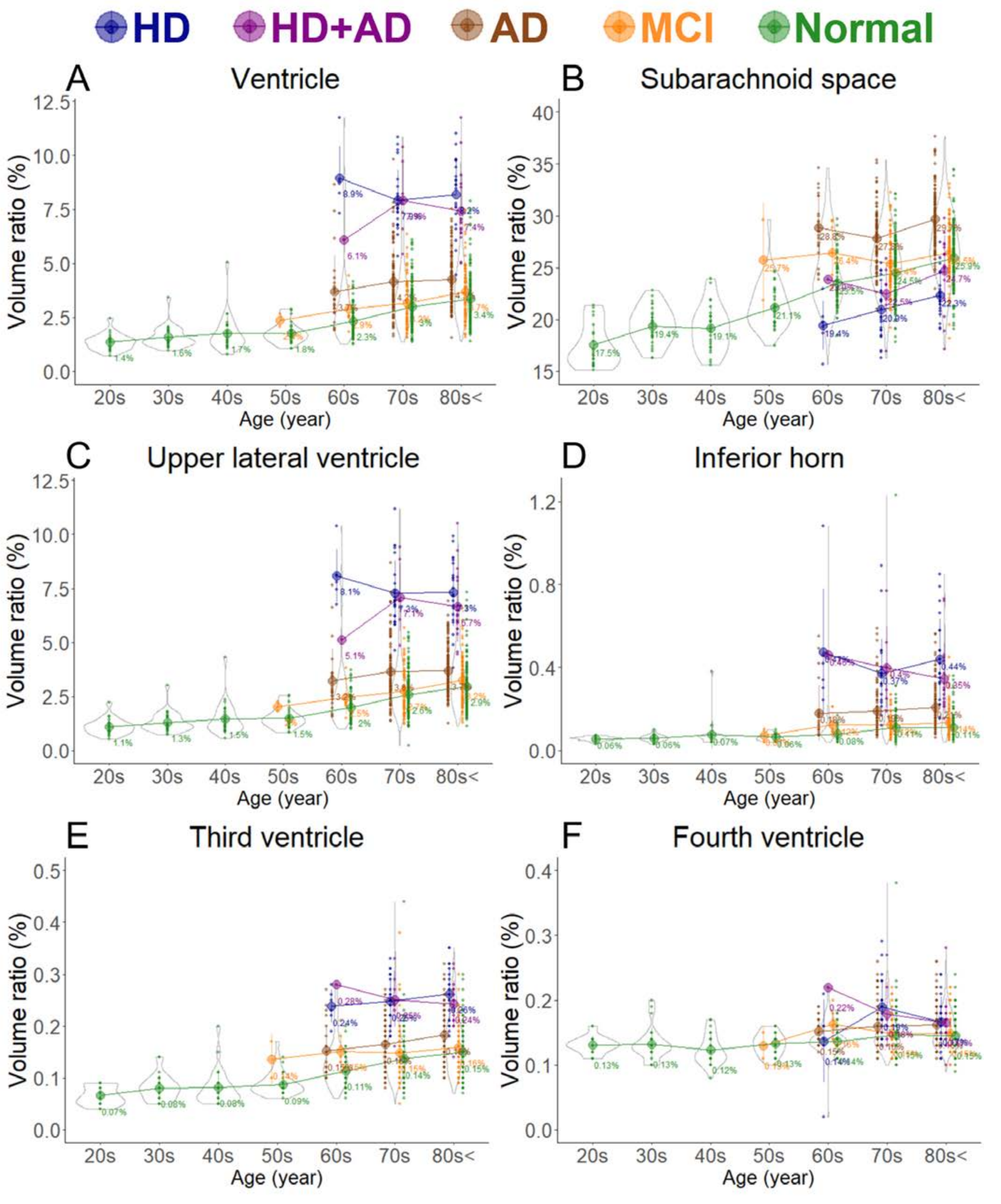

Supplementary Fig. 1. Segmented region volume ratio of cerebrospinal fluid spaces. Each graph has violin plots for the distribution of the segmented volume ratio and a circle with line graphs for the mean volume ratio in each decade stratified according to disease. Dark blue indicates Hakim’s disease (HD, N=52), brown indicates Alzheimer’s disease (AD, N=256), purple indicates HD with AD (HD + AD, N=25), orange indicates mild cognitive impairment (MCI, N=163), and green indicates normal volunteers (N=474).
A: Ventricle; B: Subarachnoid space; C: Upper part of lateral ventricle; D: Inferior horn of lateral ventricle; E: Third ventricle; and F: Fourth ventricle.

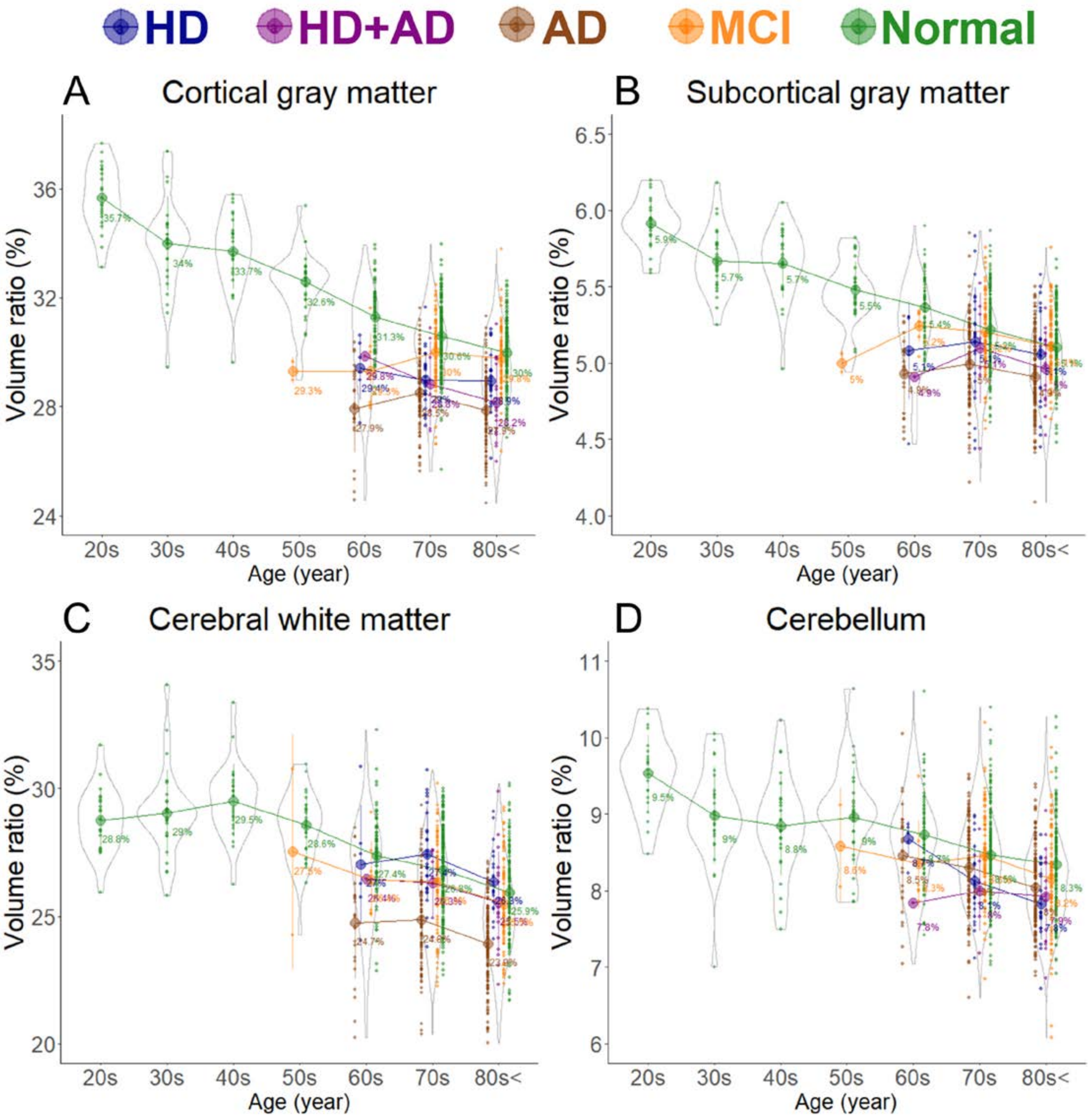

Supplementary Fig. 2. Segmented region volume ratio of large-subregions.
Each graph has violin plots for the distribution of the segmented volume ratio and a circle with line graphs for the mean volume ratio in each decade stratified according to disease. Dark blue indicates Hakim's disease (HD, N=52), brown indicates Alzheimer's disease (AD, N=256), purple indicates HD with AD (HD + AD, N=25), orange indicates mild cognitive impairment (MCI, N=163), and green indicates normal volunteers (N=474).
A: Cortical gray matter; B: Subcortical gray matter; C: Cerebral white matter; and D: Cerebellum.

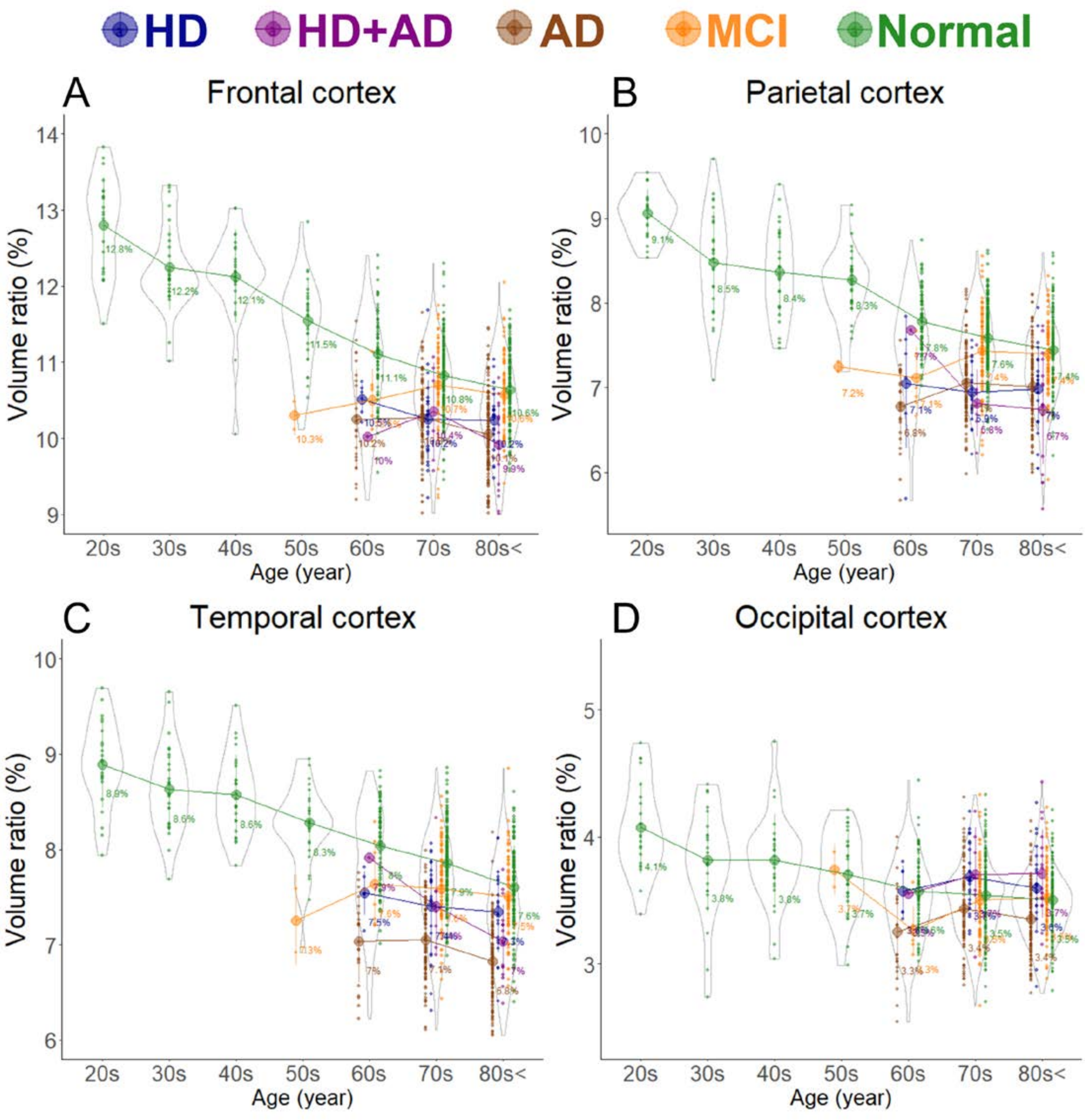

Supplementary Fig. 3. Segmented region volume ratio of mid-subregions.
Each graph has violin plots for the distribution of the segmented volume ratio and a circle with line graphs for the mean volume ratio in each decade stratified according to disease. Dark blue indicates Hakim's disease (HD, N=52), brown indicates Alzheimer's disease (AD, N=256), purple indicates HD with AD (HD + AD, N=25), orange indicates mild cognitive impairment (MCI, N=163), and green indicates normal volunteers (N=474).
A: Frontal cortex; B: Parietal cortex; C: Temporal cortex; and D: Occipital cortex.

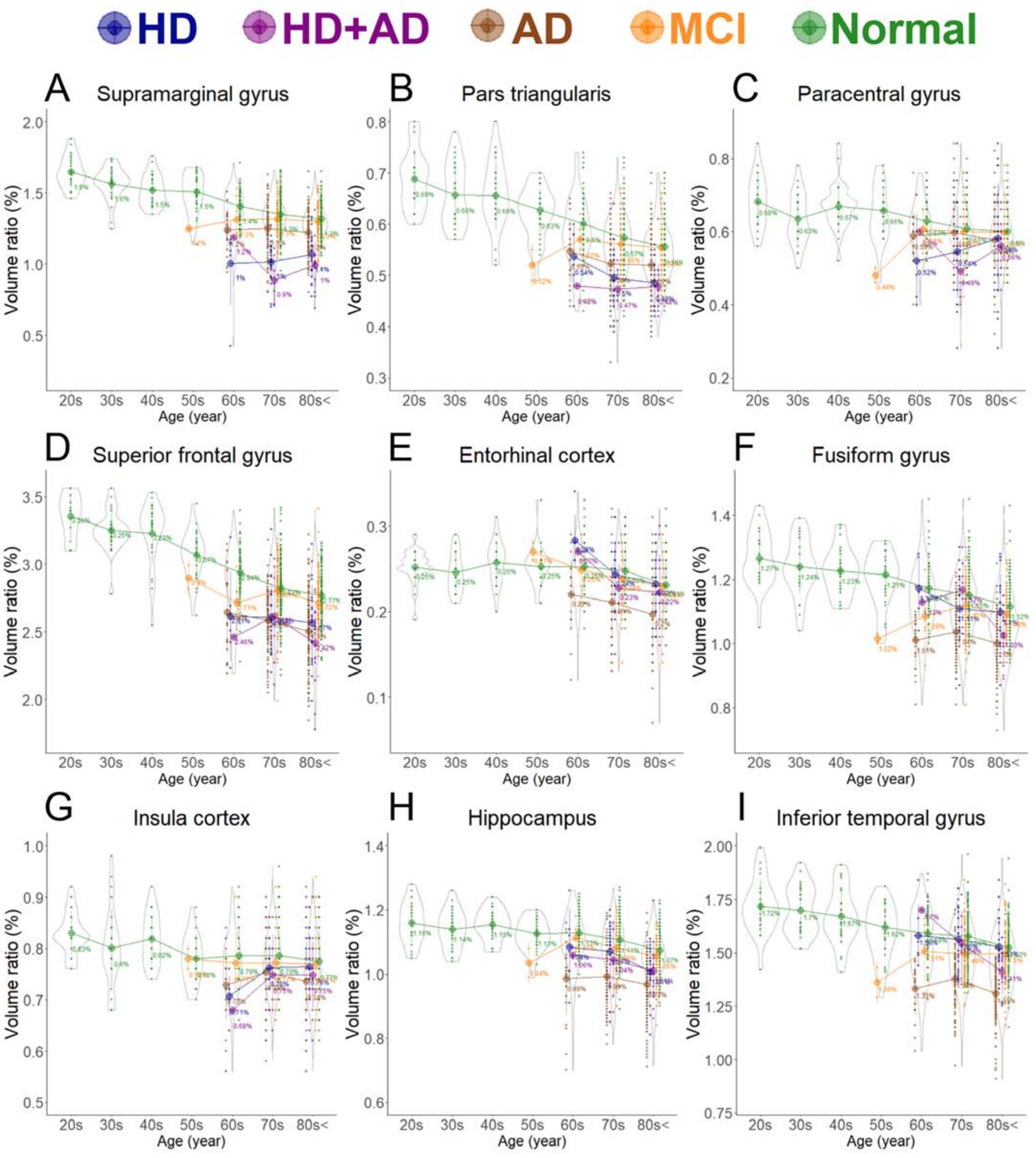

Supplementary 4. Segmented region volume ratio of specific brain subregions.
Each graph has violin plots for the distribution of the segmented volume ratio and a circle with line graphs for the mean volume ratio in each decade stratified according to disease. Dark blue indicates Hakim's disease (HD, N=52), brown indicates Alzheimer's disease (AD, N=256), purple indicates HD with AD (HD + AD, N=25), orange indicates mild cognitive impairment (MCI, N=163), and green indicates normal volunteers (N=474).
A: Supramarginal gyrus; B: Pars triangularis; C: Paracentral gyrus; D: Superior frontal gyrus; E: Entorhinal cortex; F: Fusiform gyrus; G: Insula cortex; H: Hippocampus; and I: Inferior temporal gyrus.

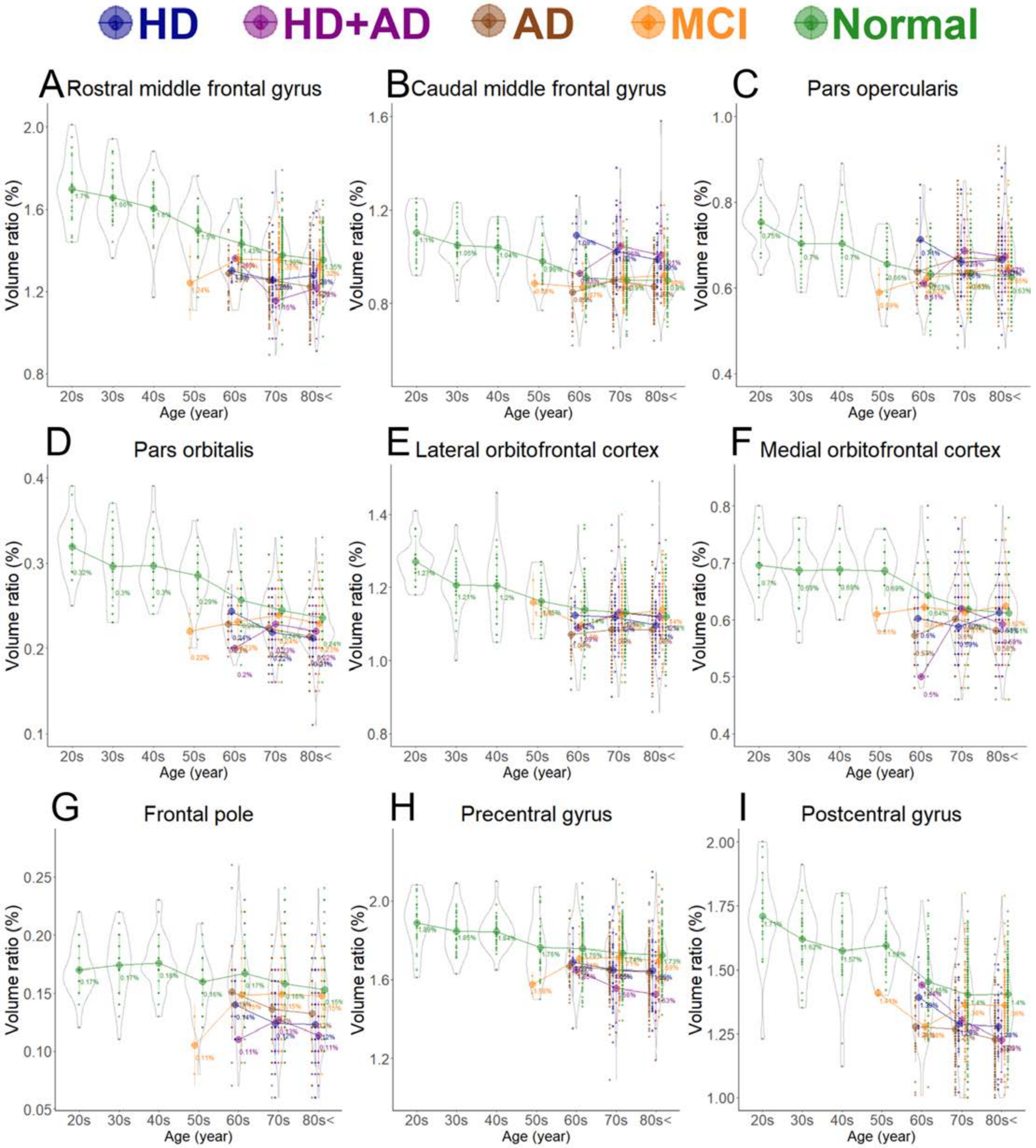

Supplementary Fig. 5. Segmented region volume ratio of specific brain subregions. Each graph has violin plots for the distribution of the segmented volume ratio and a circle with line graphs for the mean volume ratio in each decade stratified according to disease. Dark blue indicates Hakim's disease (HD, N=52), brown indicates Alzheimer's disease (AD, N=256), purple indicates HD with AD (HD + AD, N=25), orange indicates mild cognitive impairment (MCI, N=163), and green indicates normal volunteers (N=474).

A: Rostral middle frontal gyrus; B: Caudal middle frontal gyrus; C: Pars opercularis; D: Pars orbitalis; E: Lateral orbitofrontal cortex; F: Medial orbitofrontal cortex; G: Frontal pole; H: Precentral gyrus; and I: Postcentral gyrus.

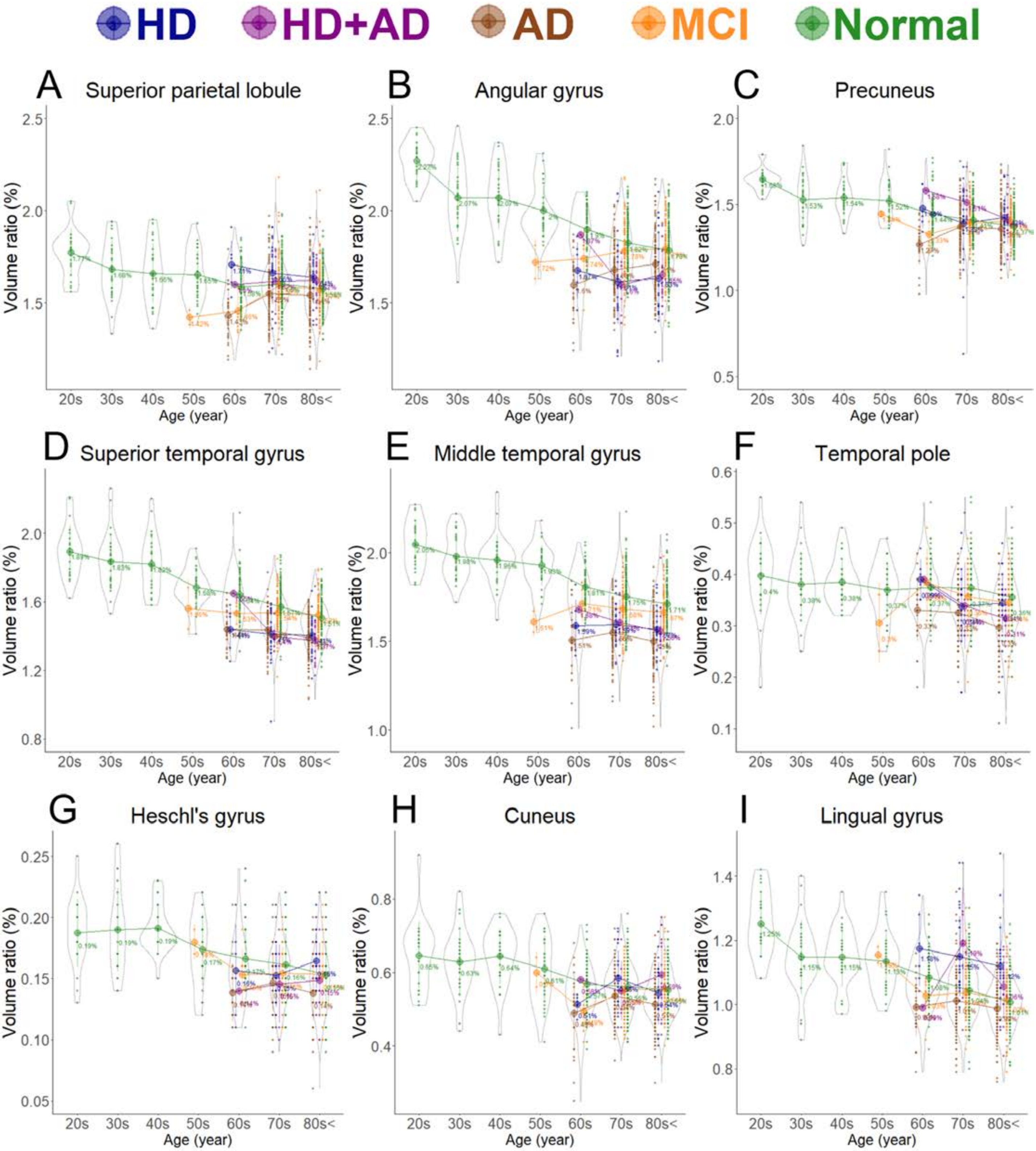

Supplementary Fig. 6. Segmented region volume ratio of specific brain subregions. Each graph has violin plots for the distribution of the segmented volume ratio and a circle with line graphs for the mean volume ratio in each decade stratified according to disease. Dark blue indicates Hakim's disease (HD, N=52), brown indicates Alzheimer's disease (AD, N=256), purple indicates HD with AD (HD + AD, N=25), orange indicates mild cognitive impairment (MCI, N=163), and green indicates normal volunteers (N=474).

A: Superior parietal lobule; B: Angular gyrus; C: Precuneus; D: Superior temporal gyrus; E: Middle temporal gyrus; F: Temporal pole; G: Heschl's gyrus; H: Cuneus; and I: Lingual gyrus.

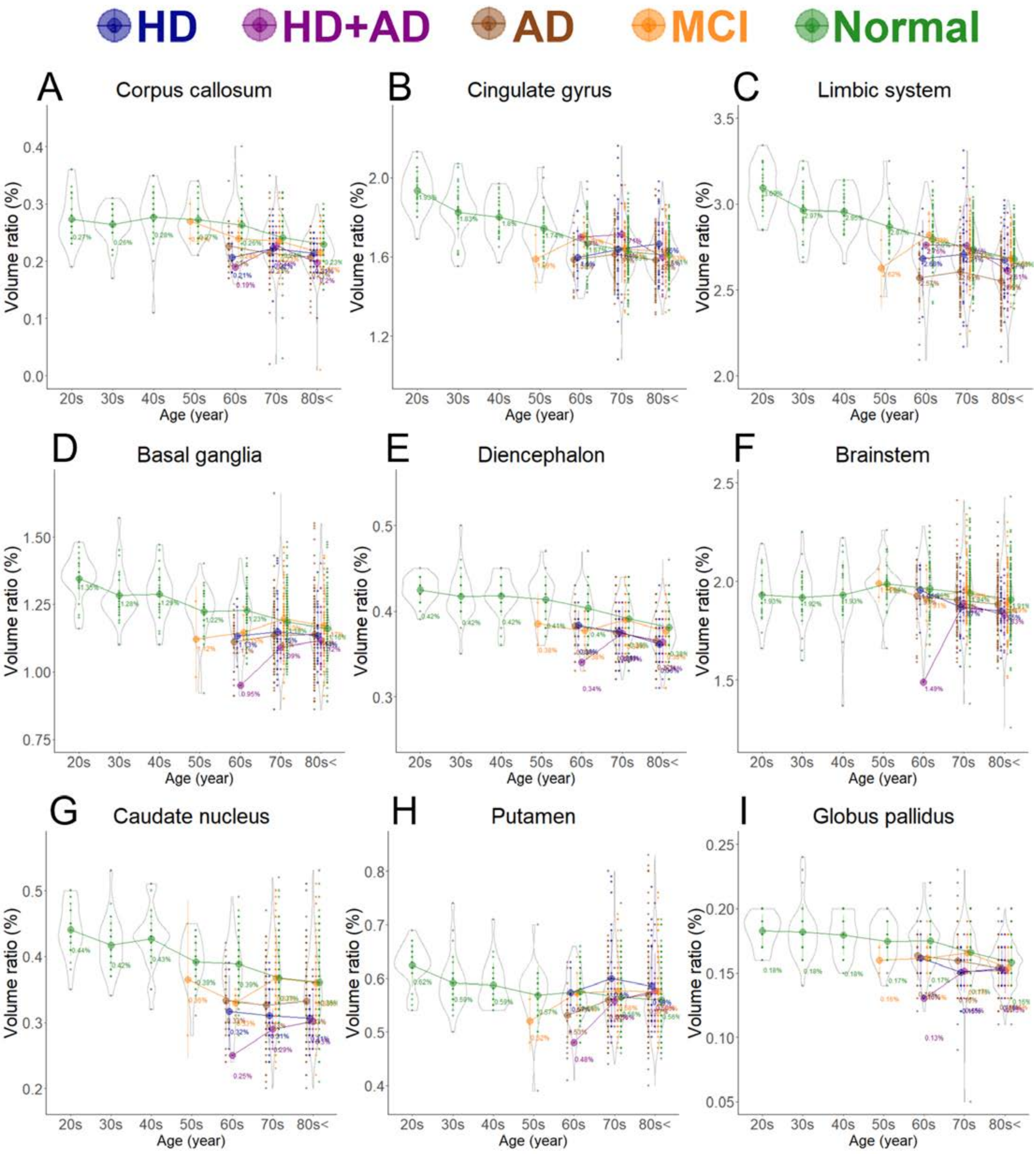

Supplementary Fig. 7. Segmented region volume ratio of specific brain subregions. Each graph has violin plots for the distribution of the segmented volume ratio and a circle with line graphs for the mean volume ratio in each decade stratified according to disease. Dark blue indicates Hakim's disease (HD, N=52), brown indicates Alzheimer's disease (AD, N=256), purple indicates HD with AD (HD + AD, N=25), orange indicates mild cognitive impairment (MCI, N=163), and green indicates normal volunteers (N=474).
A: Corpus callosum; B: Cingulate gyrus; C: Limbic system; D: Basal ganglia; E: Diencephalon; F: Brainstem; G: Caudate nucleus; H: Putamen; and I: Globus pallidus.
