# Supplementary material for: Regional brain volume changes in Hakim’s disease versus Alzheimer’s and mild cognitive impairment
Source: Brain Commun. 2025 Mar 26;7(2):fcaf122. doi: 10.1093/braincomms/fcaf122 (PMC11997787; doi:10.1093/braincomms/fcaf122)
Supplement: fcaf122_Supplementary_Data [file fcaf122_Supplementary_Data.zip › Supplementary_Table_1_legend.docx]

Supplementary Table 1: Mean volume ratios, standard deviations for 100 detailed brain subregions and 7 CSF subregions, and P-values for t-test compared with healthy controls.
