# Supplementary material for: Regional brain volume changes in Hakim’s disease versus Alzheimer’s and mild cognitive impairment
Source: Brain Commun. 2025 Mar 26;7(2):fcaf122. doi: 10.1093/braincomms/fcaf122 (PMC11997787; doi:10.1093/braincomms/fcaf122)
Supplement: fcaf122_Supplementary_Data [file fcaf122_Supplementary_Data.zip › Supplementary_video_legends.docx]

**Supplementary videos**

Video 1. 3D View of Brain Subregion Analysis in a Healthy Volunteer
Video 2. Axial View of Brain Subregion Analysis in a Healthy Volunteer
Video 3. Coronal View of Brain Subregion Analysis in a Healthy Volunteer
Video 4. Sagittal View of Brain Subregion Analysis in a Healthy Volunteer
Video 5. 3D View of Brain Subregion Analysis in a Patient with Hakim's Disease
Video 6. Axial View of Brain Subregion Analysis in a Patient with Hakim's Disease
Video 7. Coronal View of Brain Subregion Analysis in a Patient with Hakim's Disease
Video 8. Sagittal View of Brain Subregion Analysis in a Patient with Hakim's Disease
